# Supplementary material for: Sustained-input switches for transcription factors and microRNAs are central building blocks of eukaryotic gene circuits
Source: Genome Biol. 2013 Aug 23;14(8):R85. doi: 10.1186/gb-2013-14-8-r85 (PMC4054853; doi:10.1186/gb-2013-14-8-r85)
Supplement: Additional file 5 — HTML Browsable Motif Output. Zipped folder containing all WaRSwap and FANMOD motif output, viewable in a web browser. [file gb-2013-14-8-r85-S5.ZIP › HTML_browsable_motif_output/FANMOD_ath_tair9/sigs_fanmodm-2000.pvals.heatmaps.html/motif_id_38_011011002_tftype_ath_upstream_-3000_0.html]

```
BG_MODEL = FANMOD
MOTIF_ID = 38_011011002
TF_TYPE = ath
UPSTREAM = -3000_0


PVals
FN_0.2	FN_0.4	FN_0.6	FN_0.8
dg_60.genes	0	0	0.038	0.002
dg_70.genes	0	0	0	0.005
dg_80.genes	0	0	0	0.038

ZScores
FN_0.2	FN_0.4	FN_0.6	FN_0.8
dg_60.genes	5.969	6.666	1.79	2.935
dg_70.genes	5.576	6.856	4.574	2.635
dg_80.genes	5.682	7.172	4.062	1.732

StDevs
FN_0.2	FN_0.4	FN_0.6	FN_0.8
dg_60.genes	111.517	100.194	56.498	24.222
dg_70.genes	84.988	78.884	45.926	17.915
dg_80.genes	55.129	52.262	32.283	14.327
```
